# Comparison of preoperative MRI and intraoperative findings of posterior tibial tendon insufficiency

**DOI:** 10.1186/s40064-016-3114-4

**Published:** 2016-08-24

**Authors:** Matthias Braito, Martina Wöß, Benjamin Henninger, Michael Schocke, Michael Liebensteiner, Dennis Huber, Martin Krismer, Rainer Biedermann

**Affiliations:** 1 Department of Orthopedics, Medical University of Innsbruck, Anichstrasse 35, Innsbruck, Austria; 2 Department of Radiology, Medical University of Innsbruck, Anichstrasse 35, Innsbruck, Austria; 3 Department of Radiology, Medical University of Ulm, Albert-Einstein-Allee 7, Ulm, Germany; 4 Department of Experimental Orthopaedics, Medical University of Innsbruck, Innrain 36, Innsbruck, Austria

**Keywords:** Tibialis posterior tendon, Magnetic resonance imaging, Acquired adult flatfoot deformity, Posterior tibial tendon insufficiency

## Abstract

**Background:**

The purpose of this study was to investigate the radiological and surgical correlation between preoperative magnetic resonance images (MRI) and the intraoperative findings in patients with acquired adult flatfoot.

**Results:**

The overall radiological–surgical correlation between preoperative MRI and the intraoperative findings for posterior tibial tendon insufficiency was only slight to fair in our patient’s series. Comparing the most commonly used posterior tibial tendon classification systems, the classification of Rosenberg et al. and Kong et al. showed higher interobserver agreement than our modified classification system and the classification system of Conti et al.

**Conclusion:**

Further prospective studies are needed to evaluate the importance of preoperative MRI before surgical repair of posterior tibial tendon dysfunction.

## Background

Acquired adult flatfoot deformity is mainly caused by posterior tibial tendon (PTT) insufficiency (Beals et al. 1999; Trnka 2004). Treatment of PTT insufficiency depends on the stage of disease, which is usually classified according to Johnson and Strom based on clinical and radiographic findings (Myerson 1997; Johnson and Strom 1989). The use of magnetic resonance imaging (MRI) as an adjunctive diagnostic modality has been advocated especially in early stages in which the diagnosis is less clear or to exclude other related pathologies. However, while MRI is considered as the golden standard for the diagnostic workup of PTT pathologies, it’s diagnostic accuracy for PTT insufficiency has not yet been adequately studied (Chhabra et al. 2011; Rosenberg et al. 1988; Schweitzer and Karasick 2000). Many MRI abnormalities of the PTT are thought to be associated with PTT dysfunction, including both primary changes of PTT texture (e.g. insertional tendinosis, tenosynovitis, and tendon rupture) and secondary changes such as failure of the spring ligament and deltoid ligament (Chhabra et al. 2011; Balen and Helms 2001; Schweitzer and Karasick 2000; Khoury et al. 1996; Narvaez et al. 1997). These MRI features may be further categorized into imaging-based classification systems to assist in the treatment decision process (Kong and Van Der Vliet 2008; Rosenberg et al. 1988; Conti et al. 1992). The most commonly used MRI-imaging systems of PTT insufficiency compromise the classifications systems suggested by Rosenberg et al. (1988), Conti et al. (1992), and Kong and Van Der Vliet (2008). To our knowledge, the predictability of these MRI classification systems with regard to intraoperative findings in surgery performed for PTT insufficiency has not been compared so far. Therefore, the purpose of this study was to retrospectively investigate the radiological–surgical correlation between preoperative MRI (categorized into above mentioned classification systems) and intraoperative findings in our patient’s series with acquired adult flatfoot deformity.

## Results

### Study population

The study population consisted of three men (13.6 %) and 19 women (86.4 %). Surgery was performed at a mean age of 53.3 years (23–71 years) on seven right (31.8 %) and 15 left (68.2 %) ankles. 16 patients (72.7 %) were treated by flexor digitorum longus transfer and medial displacement calcaneal osteotomy alone, six patients (27.3 %) required additional procedures (medial Lisfranc arthrodesis, arthrodesis of the first MTP joint, bunionectomy, claw toe correction).

### Interobserver agreement of tested classification systems

The detailed results of our investigation (Table 1) showed slight to fair interobserver agreement (Table 2) between preoperative MRI findings (MR1, MR2) and intraoperative findings (OP1, OP2). Cohen’s kappa coefficients were higher for the classification systems by Rosenberg et al. (M2; kappa = 0.33, CI 0.32–0.35) and Kong et al. (M4; kappa = 0.33, CI 0.32–0.33), whereas our modified classification system (M1; kappa = 0.08, CI 0.05–0.10) and the classification system by Conti et al. (M4; kappa = 0.17, CI 0.15–0.18) showed lower interobserver agreement. Interobserver agreement was fair to moderate (kappa = 0.32–0.52) between the preoperative MRI findings of the two radiologists with the highest agreement found with the classification system by Kong and Van Der Vliet (2008).

**Table 1:** Results of the tibialis posterior tendon appearance classified by the 4 investigators (OP1, OP2, MR1, MR2) and 4 methods (M1–4)

Pat. ID	OP1				OP2				MR1				MR2			
	M1	M2	M3	M4	M1	M2	M3	M4	M1	M2	M3	M4	M1	M2	M3	M4
1	IIIb	II	II	II	IIIb	II	IIIA	II	IIIa	I	IB	I	IIIb	II	II	II
2	II	I	IA	I	II	I	II	I	IIIb	I	II	I	II	I	*NA*	I
3	IIIb	II	II	II	IIIb	II	IIIA	II	IIIa	I	IA	I	I	*NA*	*NA*	*NA*
4	IIIb	II	IIIA	II	IIIa	I	IIIA	I	IIIa	I	IB	I	IIIa	I	IB	I
5	IVa	III	IIIA	III	IVa	I	IIIA	I	IVb	III	IIIB	III	IVa	III	IIIB	III
6	II	I	IB	I	II	I	IB	I	IVa	II	IIIA	II	IIIa	I	IA	I
7	II	I	IB	I	IIIa	I	IB	I	IIIb	II	II	I	II	I	IB	I
8	II	I	IA	I	II	II	II	II	IIIb	II	II	II	II	I	IA	I
9	IVa	III	IIIB	III	IVb	III	IIIB	III	IVb	III	IIIB	III	IVb	III	IIIB	III
10	II	I	IA	I	II	I	II	II	IIIa	I	II	I	IIIa	II	II	II
11	I	I	IA	I	II	I	IA	I	II	I	IB	I	II	I	*NA*	I
12	IVb	III	IIIB	III	IVb	III	IIIB	III	II	I	IB	I	II	I	I	I
13	IVb	III	IIIB	III	IVb	III	IIIB	III	IIIb	II	II	II	IIIb	II	IIIA	II
14	IVa	III	IIIB	III	IVa	III	IIIB	III	IVb	III	IIIB	III	IVb	III	IIIB	III
15	II	I	IA	I	II	I	II	II	II	I	I	I	I	*NA*	*NA*	*NA*
16	IVa	III	IIIB	III	IVa	III	IIIB	III	IVb	III	IIIB	III	IVb	III	IIIB	III
17	IIIb	II	II	II	IIIb	II	II	II	IIIa	II	II	II	IIIa	I	IA	I
18	IIIb	II	II	II	IIIb	II	II	II	IIIa	II	II	II	IIIa	I	IA	I
19	IIIa	II	II	II	IIIb	II	II	II	IIIb	II	IIIA	II	IIIb	II	II	II
20	IIIa	II	II	II	IIIb	II	II	II	IIIa	II	IB	I	II	I	I	I
21	IIIa	II	IB	II	II	I	IA	I	IIIa	I	II	I	IIIa	I	II	I
22	II	I	IB	I	II	I	IB	I	IIIb	II	IIIA	II	IIIb	II	IIIA	II

**Table 2:** Interobserver agreement (Cohen’s kappa coefficient) of tested classification systems

Classification	M1	M2 (Rosenberg et al. 1988)	M3 (Conti et al. 1992)	M4 (Kong and Van Der Vliet 2008)
Intraoperative findings				
OP1–OP2	0.59	0.72	0.60	0.59
Preoperative MRI findings				
MR1–MR2	0.48	0.39	0.32	0.52
Intraoperative–preoperative findings				
OP1–MR1	−0.05	0.30	0.07	0.30
OP1–MR2	0.10	0.32	0.24	0.32
OP2–MR1	0.05	0.43	0.17	0.30
OP2–MR2	0.21	0.28	0.18	0.38
OP–MR (mean kappa coefficient, 95 % CI)	0.08 (0.05–0.10)	0.33 (0.32–0.35)	0.17 (0.15–0.18)	0.33 (0.32–0.33)

## Discussion

The overall radiological–surgical correlation between the preoperative MRI and the intraoperative findings in PTT insufficiency was only slight to fair in our patient’s series. Comparing the most commonly used PTT classification systems, the classification of Rosenberg et al. (1988) and Kong and Van Der Vliet (2008) showed higher interobserver agreement than our modified classification system and the classification system of Conti et al. (1992).

Our study had several limitations that might have influenced our findings: We hypothesize that the poor correlation between MRI and intraoperative findings of the PTT in our study might be explained by three main reasons: The time interval between the preoperative MRI and surgery averages 4 months (range 14 days to 10 months). Therefore, progressive deterioration of the PTT might have influenced our findings. Second, as a consequence of the retrospective study design, the description of the PTT appearance in the surgical reports was not standardized. Some descriptions of the PTT tendon were imprecise and allowed more freedom in classification than the MRI findings. Nevertheless, interobserver agreement between OP1 and OP2 was still moderate to substantial (kappa = 0.58–0.72). Classification systems with more selection possibilities showed poorer interobserver correlation, whereas the classification systems of Rosenberg et al. (1988) and Kong and Van Der Vliet (2008) showed higher interobserver agreement. Third, differences between the MRI protocol of our department and other radiologic imaging centers might have influenced our interpretations.

Our findings are fairly consistent with previous studies that evaluated PTT dysfunction by MRI. Rosenberg et al. (1988) classified PTT tears in three types and report an overall accuracy of 73 % of MRI for detecting PTT tears. Based on their findings, the classification system of Conti et al. (1992) further subdivides partial PTT tears depending on the size of the abnormal tendon signal intensity. In accordance with our results, a lower overall correlation of 40 % between MRI and surgical classification was found in that study. The authors attributed this finding to the fact that intratendinous degeneration might not be visible during surgical inspection. PTT degeneration might also present apparently normal on MRI and partial PTT disruptions might not be visible on MRI (Schweitzer and Karasick 2000). On the other hand, irregularities of the PTT surface at the center of the chiasma crurale (Buck et al. 2010), at the medial malleolus (magic angle artifact), and at the complex distal PTT insertion (Pastore et al. 2008; Fernandes et al. 2006) might be misinterpreted as tendon degeneration or rupture. Tendon inhomogenity of the PTT must generally be interpreted with caution, since Perry et al. (2003) found pain intensity to be correlated with tendon and peritendon enhancement but not with tendon inhomogeneity. Kong and Van Der Vliet (2008) considered MRI as gold standard for evaluation of PTT dysfunction. The correlation of MRI interpretation between their two radiologists was found to be highest for the classification system suggested by these authors and the classification system of Rosenberg et al. (1988). Khoury et al. (1996) found a high correlation of MRI abnormalities and intraoperative findings in eleven patients operated for PTT dysfunction. However, they assumed an overlap of MRI findings in cases of severe PTT tendinosis and partial PTT tearing. The authors further stressed the use of oblique axial planes to evaluate the tendon’s cross section behind the medial malleolus. A high sensitivity of 94 % but low specificity of 6 % for detection of Achilles and posterior tibial tendon tears by preoperative MRI was confirmed by the findings of Kuwada (2008).

The use of advanced imaging before surgical repair of PTT dysfunction is still subject to discussion (Baca et al. 2014): The use of MRI seems to be advantageous especially in early stages of the disease and unclear conditions, as MRI allows evaluation of the tarsal tunnel (Erickson et al. 1990), the distal tendon insertion in case of accessory navicular bone (Kiter et al. 1999), and secondary signs of PTT dysfunction such as tibial spurs, subtendinous bone edema, unroofing of the talus and tendon (sub-) luxation (Schweitzer and Karasick 2000). In addition, associated pathologies such as spring ligament (Yao et al. 1999; Williams et al. 2013) and sinus tarsi abnormalities are seen on MRI especially in advanced PTT dysfunction and could then be addressed during surgery (Balen and Helms 2001; Shibuya et al. 2008). For these indications, the use of MRI partially competes with other imaging modalities. Sonography has been compared to MRI and showed consistent results in 77 % of cases (Nallamshetty et al. 2005; Lhoste-Trouilloud 2012; Hamel and Seybold 2002). Furthermore, PTT tenography and local anaesthetic tendon sheath injections were described as reliable diagnostic tools (Cooper et al. 2007; Jaffee et al. 2001). Recently, the use of tendoscopy has yielded diagnostic advantages for early recognition of PTT dysfunction (Gianakos et al. 2015).

## Conclusions

In conclusion, our results did not show a high correlation between preoperative MRI and surgical findings for PTT insufficiency. Since interpretation of our results is limited by the retrospective study design, further prospective studies are necessary to evaluate the value of preoperative MRI for the treatment of PTT insufficiency.

## Methods

The study has been approved by the local ethics committee (Medical University of Innsbruck) and has been performed in accordance with the ethical standards laid down in the 1964 Declaration of Helsinki and its later amendments. All persons gave their informed consent prior to their inclusion in the study.

### Study population

The patient population in this retrospective analysis consisted of a consecutive series of 130 patients that were treated for adult-acquired-flatfoot/PTT insufficiency at our department between January 2000 and December 2013. Exclusion criteria were defined as follows: (1) treatment for adult-acquired-flatfoot without surgical exploration of the PTT or missing description of intraoperative tendon appearance in the surgical report (n = 101) and (2) missing preoperative MRI scan (n = 7). Consequently, 22 patients that had received a tibialis posterior tendon tenosynovectomy or reconstruction by one of three consultant orthopaedic surgeons at our department were included in the study.

### Method and setting of data collection

The surgical reports of all included patients were analyzed by two experienced orthopaedic registrar-grade surgeons (OP1, OP2) and the preoperative MRI scans of all included patients were analyzed by two consultant radiologists specialized in musculoskeletal MRI (MR1, MR2). None of the four investigators (OP1, OP2, MR1, MR2) was involved in the patients’ treatment and all investigators were blinded to other radiographic findings or patients’ history to avoid measurement bias. The tibialis posterior tendon appearance was classified according to four different classification methods (M1–4; Tables 3, 4, 5, 6) by each investigator: (1) our modified classification system based on the classification systems of Rosenberg et al. (1988) and Lee et al. (2005), (2) the original classification system described by Rosenberg et al. (1988), (3) the classification system of Conti et al. (1992), and the classification system described by Kong and Van Der Vliet (2008). The modified classification system was introduced to further specify the tendon condition in partial and complete ruptures of the PTT in order to remedy this deficit of other classification systems.

**Table 3:** Our modified classification system

Type	Description	
I	Tendon without abnormalities	
II	Peritendinitis, tendinitis without tears, elongation of the tendon, degenerative changes of the tendon	
III	Partial tendon lesion	
	IIIa	With vertical splits, partial tears and lesions, potential thickening of the tendon
	IIIb	Thinning of the tendon, but in general consistently with potential swelling of the tendon in the distal part of the thinning
IV	Complete tendon tear with signs to repair	
	IVa	Tendon tissue linked with an insufficiently cicatricial tissue
	IVb	Tendon gap
	IVx	Classification not clearly attributable

**Table 4:** Classification system by Rosenberg et al.

Type	Description
I	Partially torn bulbous tendon with vertical splits and defects
II	Partially torn, attenuated tendon
III	Complete rupture with a tendon gap

**Table 5:** Classification system by Conti et al.

Type	Description
I	
IA	One or two fine longitudinal splits in the posterior tibial tendon without evidence of intrasubstance degeneration. The splits are frequently found on the undersurface of the tendon
IB	Increased number of longitudinal splits with an increase in tendon width with mild surrounding fibrosis. There is no significant tendon degeneration
II	The posterior tibial tendon is narrowed, with long longitudinal splits and intramural degeneration. Often, the tendon has a bulbous appearance distal to the attenuated portion
III	
IIIA	This is notable for more diffuse swelling of the posterior tibial tendon, with uniform degeneration becoming a prominent feature. There are a few strands of intact tendon through the area of degeneration
IIIB	There is a complete rupture of the posterior tibial tendon, with complete replacement of the tendon by scar tissue

**Table 6:** Classification system by Kong et al.

Type	Description
I	Partial tear: fusiform enlargement, intrasubstance degeneration, longitudinal split
II	Partial tear: stretching and elongation
III	Complete tear: discontinuity

### Image protocol

MRI was performed on a 1.5-T system (Magnetom Avanto or Symphony Vision, Siemens, Germany) at our department or external radiologic centre. Patients were scanned in the supine position. In five patients a dedicated ankle coil and in 17 patients a knee coil was used. The MR imaging protocol included the following sequences in at least one orientation: T1-weighted TSE images, T2-weighted TSE images, Short-Tau-Inversion-Recovery (STIR) or PD-weighted images with fat-saturation (fluid-sensitive sequences). All images were performed with 3 mm slice-thickness. Additionally a DESS (Dual Echo Steady state) sequence was performed in five patients and in nine patients a T2 MEDIC 3D (Multi Echo Data Image Combination) sequence was performed, both in sagittal orientation.

### Analysis of MR images

MR images were read in consensus by two radiologists (MR1, MR2; both with over 7 years of experience in reading MRI of the musculoskeletal system) at a workstation with the Impax 6 (Agfa Healthcare, Mortsel, Belgium) picture archiving and communication system (PACS). The presence of tendon abnormality including tendinosis, tenosynovitis, low- and high-grade partial tear, and complete tear was registered after evaluation of the full length of the tendon. Tendinosis was defined as irregularity of the tendon contour and/or intrasubstance intermediate signal in fluid-sensitive sequences (in multiple planes) and/or thickening of the PTT tendon (greater than twice the size of the flexor digitorum longus tendon), and tenosynovitis was defined as the presence of circumferential fluid within the synovial tendon sheath greater than 2 mm in maximal width. Low-grade partial tear was defined as an intrasubstance area of high signal in fluid-sensitive sequences, with or without extension to the tendon surface. High-grade partial tear and complete tear were defined as near full thickness or full thickness discontinuity of the tendon fibers, respectively. Based on these findings the PTT appearance was classified according to four different classification methods.

### Statistics

Statistical analysis was performed using SPSS (version 21.0, IBM Corporation, Armonk, New York, United States). The interrater agreement for the classified tibialis posterior tendon appearance described in the surgical report (OP1, OP2) and the appearance in the preoperative MRI scan (MR1, MR2) was analyzed for each classification system (M1–4) using Cohen’s kappa coefficient. Furthermore, interrater agreement between the 2 orthopaedic and the 2 radiological investigators was calculated. The strength of the interrater agreement was considered as poor (kappa < 0), slight (kappa 0.01–0.20), fair (kappa 0.21–0.40), moderate (0.41–0.60), substantial (0.81–0.80), and almost perfect (kappa > 0.80) according to Landis and Koch (1977).

## Abbreviations

MRI: magnetic resonance imaging
PTT: posterior tibial tendon
